# Chronic Hydrocephalus in a Horse

**Published:** 1899-01

**Authors:** Wilfred Lellman

**Affiliations:** Professor of Parasites, Parasitic and Canine Diseases, New York College of Veterinary Surgeons


### REPORTS OF CASES.

### CHRONIC HYDROCEPHALUS IN A HORSE.[1]

By Wilfred Lellman, V.M.D.,
PROFESSOR OF PARASITES, PARASITIC AND CANINE DISEASES, NEW YORK COLLEGE OF VETERINARY SURGEONS.

On August 1st of this year I was requested to examine a horse which had been sick for several months. The owner told me that the animal had been treated by several veterinarians, but without success. The owner also told me that the diagnoses of the veterinarians differed altogether. The history of the case did not amount to anything.

Bay gelding, trotter, eight years old, with a record of 2.30. The animal is in a rather poor condition; lustre of the hair somewhat dull; skin very scaly; visible mucous membranes of normal rose-color. The pulse is rather soft, somewhat irregular, subnormally slow, beating twenty-eight to thirty-two times per minute. The temperature per rectum normal. Auscultation of the heart-region reveals arhythmic heart-beating, with intermission of three or four heart-shocks; the first heart-sound appears not quite distinct, rather lower than normal, while the second is rather abnormally strong; venous pulse quite high. Respiration is performed nine times per minute, the horse taking off and on a deep breath.

Thorough examination of the digestive tract reveals slow, peristaltic movements, feces being small-balled, containing undigested grains of oats and being covered with an abnormally thick coat of mucus. The appetite appears to be capricious.

Examination shows the urine to be of remarkably low specific gravity (1008), of light color, and rather watery; the quantity, however, seems to be within normal limits. It is slightly acidulated, which is most probably due to chronic intestinal catarrh. Albumin and sugar are not present. Microscopical examination does not reveal anything important.

Examination of the eyes shows considerably dilated pupils. By means of an ophthalmoscope I find the optic papilla rather anæmic and somewhat atrophied. When watching the animal closely I noticed that the attentiveness is not normal; also, that the eye is

[1] Read before the December meeting of the Veterinary Medical Association of New York County.

not bright, being rather without expression, somewhat stupid, though when calling him the horse will lift his head and listen.

When at his feed the horse performs mastication rather slowly and with frequent intermissions.

In order to make the examination perfect I had the horse hitched up and put him before a wagon; then I gave him a good drive from down-town (Ninth Street) up to Eighty-second Street. There I noticed a pronounced attack of vertigo; I had to stop for several minutes until he got over it.

I drove the horse back and examined him thoroughly again after having him brought to a perfectly quiet place. I watched the horse for several minutes. It appeared to be drowsy, paying no attention to the surroundings; off and on moving the ears irregularly, the head being dependent. I put my hand into his ears, a manipulation which the animal did not object to at all. Thereupon I lifted the right leg and crossed the same over the left one in front, then putting the right leg to the floor, so that the horse stands on its front legs in a crossed position. In this position I kept the horse for a minute and a half, in which time it never tried to retake the normal position. I stepped several times on the coronet, the animal not pulling its legs from under. After this examination I took the horse by the bridle and talked to him, trying to back him up. He showed only slow reaction, and it was rather hard to back him up. The examination took about half an hour. During this time the respiration had become perfectly normal, the pulse beating thirty-two times per minute. The pupils appeared considerably dilated.

According to the result of my examination, I was sure about my diagnosis, which was chronic hydrocephalus, including the possibilities of either cholesteatomata of the vascular plexuses or some other tumor, or a parasite or exostosis on the internal surface of one of the cranial bones.

I told the owner my diagnosis and also the prognosis. I explained to him that the horse might stay this way for years without getting worse; that during cooler weather an improvement might show itself; but that, on the other hand, the condition of the animal might all at once become very serious, so that he could not run the risk of driving the horse any longer. The owner took my diagnosis with much disappointment, as I noticed; he wanted me to do something for the animal to improve his appetite and condition, so I gave the horse a tonic combined with stomachica. The appetite improved, and also after four weeks I could notice a slight improvement in his condition. Several times during these four weeks I examined the urine of the animal, and found it of low specific gravity; sometimes of higher specific gravity. In the course of those changes I distinctly noticed a difference in the clinical symptoms, they being less pronounced always when the urine showed low specific gravity. This could only be derived from circulatory troubles within the kidneys, most probably due to functional troubles of certain vasomotor centres. About five weeks after my first examination I was requested to call as quickly as possible, as the horse had dropped down and was not able to get on its legs. This sudden change was rather to my satisfaction. When I arrived the stableman told me that while he was walking the horse, all at once, as he said, the animal got on its hind legs, made a dive through the air, and landed on its front knees and head; he had made several trials since to get on his legs, but was unable to do so. The horse was lying on the right side. After I had satisfied myself that no fracture of any bone was there I proceeded to follow the regular course of examination. Temperature of the cranium considerably increased; temperature per rectum 103° F., yet pulse beating only thirty-eight times; pupils at maximum dilation; look is staring, without expression; stupefied. Very high pulse of the jugular vein, however negative; heart-shock is arhythmic. the animal showing off and on regular convulsions. I injected hypodermically small doses of camphor and ether and ordered continuous cooling of the head with ice-water. When I called back after two hours the horse was on his legs. He was put in a sling, being very drowsy and apparently weak on its front legs. I drew off the urine, which was rather concentrated and of comparatively high specific gravity; it contained small quantities of albumin. However, the animal was able to walk to his box the same night without help. For the next four days the horse showed symptoms similar to those of an acute leptomeningitis; the temperature being between 104°–105° F.; the cranium being very hot, head lowered down almost to the floor, standing most of the time in one corner, off and on running alongside the boards of the box-stall.

I knew that it was not a case of acute leptomeningitis, but only an acute paroxysm, which is quite frequently to be observed during chronic hydrocephalus. This paroxysm occurred during the hot spell of September. No medicine was administered, the treatment consisting only in cooling the head and keeping the horse in a dark box-stall and on light feed. The horse got over these acute symptoms all right, but in general the clinical symptoms of chronic hydrocephalus became far more pronounced. During the next few weeks I had the occasion to watch the animal several times, and I saw when it was going to lie down it would first crouch on its hind legs, like a dog, and then throw himself over sideways, then lying perfectly quiet. While eating the mastication was always performed slowly and with long intermissions. Quite frequently the animal would stand there for a long time, the head down, the mouth filled up with hay, not chewing at all. Several times I found the animal standing with its head down, the extremities being in a most abnormal position, either all four legs gathered under the body or even the front extremities crossed.

I advised the owner to have the horse destroyed, as neither he nor anybody else could run the risk to sit behind him. The owner did not seem to like this idea. He still thought I was mistaken. I then told him if my diagnosis was wrong I was willing to pay for the horse. He decided to call on two disinterested professional men—one being a veterinarian, the other being a physician—a well-known pathologist. The horse was poisoned with cyanide of potash, the brains were carefully taken out, and the correctness of my diagnosis was confirmed.

Post-mortem revealed the following alterations of the brain: atrophy of the cortical substance of the hemispheres, considerable dilatation of the lateral ventricles and also of the third ventricle, containing about 15 cm. of serous fluid. Microscopically I found principally atrophy of the ganglia cells; on some places proliferation of glia cells; on others commencing fatty degeneration of the glia substance, desolation of fine bloodvessels, infiltration of perivascular lymph-sinuses, with plenty of emigrated leucocytes.

---

The Senate agricultural bill contains a clause authorizing the Secretary of Agriculture to inspect imported articles dangerous to health, and authorizes the Secretary of the Treasury to exclude such articles. A resolution has also been introduced in the Senate requiring the Committee on Agriculture to investigate the proposed German legislation against American sausages and meats, and if such measures become a law to report a bill immediately requiring the inspection of sugars, meats, wines, and all other food-products which are imported into this country from the empire of Germany.

